# Group II intron splicing factors in plant mitochondria

**DOI:** 10.3389/fpls.2014.00035

**Published:** 2014-02-18

**Authors:** Gregory G. Brown, Catherine Colas des Francs-Small, Oren Ostersetzer-Biran

**Affiliations:** ^1^Department of Biology, McGill UniversityMontreal, QC, Canada; ^2^ARC Centre of Excellence in Plant Energy Biology, The University of Western AustraliaCrawley, WA, Australia; ^3^Department of Plant and Environmental Sciences, The Alexander Silberman Institute of Life Sciences, The Hebrew University of JerusalemJerusalem, Israel

**Keywords:** group II intron, splicing, maturase, splicing factor, respiration, mitochondria, plant

## Abstract

Group II introns are large catalytic RNAs (ribozymes) which are found in bacteria and organellar genomes of several lower eukaryotes, but are particularly prevalent within the mitochondrial genomes (mtDNA) in plants, where they reside in numerous critical genes. Their excision is therefore essential for mitochondria biogenesis and respiratory functions, and is facilitated *in vivo* by various protein cofactors. Typical group II introns are classified as mobile genetic elements, consisting of the self-splicing ribozyme and its intron-encoded maturase protein. A hallmark of maturases is that they are intron specific, acting as cofactors which bind their own cognate containing pre-mRNAs to facilitate splicing. However, the plant organellar introns have diverged considerably from their bacterial ancestors, such as they lack many regions which are necessary for splicing and also lost their evolutionary related maturase ORFs. In fact, only a single maturase has been retained in the mtDNA of various angiosperms: the *matR* gene encoded in the fourth intron of the NADH-dehydrogenase subunit 1 (*nad1* intron 4). Their degeneracy and the absence of cognate ORFs suggest that the splicing of plant mitochondria introns is assisted by *trans*-acting cofactors. Interestingly, in addition to MatR, the nuclear genomes of angiosperms also harbor four genes (*nMat 1-4*), which are closely related to maturases and contain N-terminal mitochondrial localization signals. Recently, we established the roles of two of these paralogs in Arabidopsis, nMAT1 and nMAT2, in the splicing of mitochondrial introns. In addition to the nMATs, genetic screens led to the identification of other genes encoding various factors, which are required for the splicing and processing of mitochondrial introns in plants. In this review we will summarize recent data on the splicing and processing of mitochondrial introns and their implication in plant development and physiology, with a focus on maturases and their accessory splicing cofactors.

## Plant mitochondrial genomes (mtDNAs)

Mitochondria in plants house the oxidative phosphorylation (OXPHOS) machinery and many other essential metabolic pathways (for review see Millar et al., 2011). The vast majority of the proteins responsible for these processes, as well as those that participate in the biogenesis of the organelle (e.g., the translocons involved in protein import) are encoded in the nuclear genome. A small number of essential proteins, however, are encoded in the organelle's own genome. In vascular plants these genomes (mitochondrial DNAs, or mtDNAs) are much larger and more variable in size than the mtDNAs of other organisms and also display an array of other unique features (Knoop, 2012). The mtDNAs in plants encode tRNAs, rRNAs, ribosomal proteins, subunits of the respiratory machinery, including NADH:ubiquinone oxidoreductase (complex I), the cytochrome *bc*_1_ complex (complex III), cytochrome *c* oxidase (complex IV), ATP-synthase (complex V), and several other proteins involved in cytochrome *c* biogenesis and the twin-arginine protein translocation (Unseld et al., 1997; Kubo et al., 2000; Adams et al., 2002; Notsu et al., 2002; Handa, 2003; Clifton et al., 2004; Ogihara et al., 2005; Sugiyama et al., 2005). The mitochondrial translation machinery and energy transduction complexes are composed of both nuclear and organellar encoded subunits, thus necessitating complex mechanisms for the coordination of the expression of these two physically distinct genomes.

**Figure 1:**
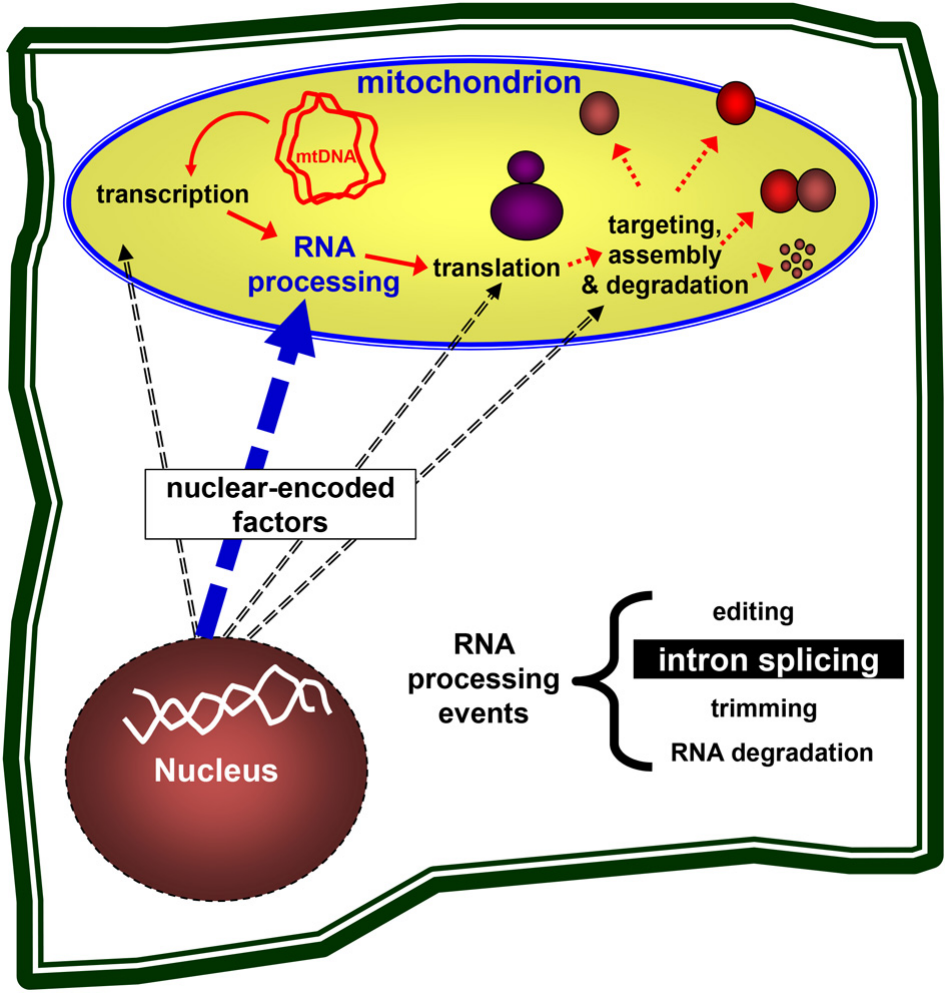
**Nuclear genes control the mitochondria biogenesis**. Nuclear-encoded genes are necessary for the expression of mitochondrial genes, including transcription, pre-mRNA processing, translation of the mRNAs into mitochondrial proteins, the assembly of ribosomes and respiratory complexes, and are also required for the targeting and degradation of organellar subunits. Plant organelles are excellent systems to study these processes in eukaryotes.

The expression of mitochondrial genes in plants is regulated primarily at the post-transcriptional level (Finnegan and Brown, 1990; Binder and Brennicke, 2003). The primary transcripts in plant mitochondria undergo extensive processing, including the nucleolytic maturation of 5' and 3' termini, RNA editing (C-to-U changes in angiosperms), and the splicing of numerous introns which lie within genes encoding proteins required for both organellar gene expression and respiration (Gagliardi and Binder, 2007; Bonen, 2008; Takenaka et al., 2008). These processes are necessary for these RNAs to carry out their functions in protein synthesis and are accomplished largely by nuclear-encoded factors, which may also provide a means to link organellar functions with environmental and/or developmental signals (see Figure 1).

In this review, we will summarize recent progress on the splicing of mitochondrial group II introns in angiosperms, with emphasis on RNA maturases and several other accessory factors.

## Group II introns

**Figure 2:**
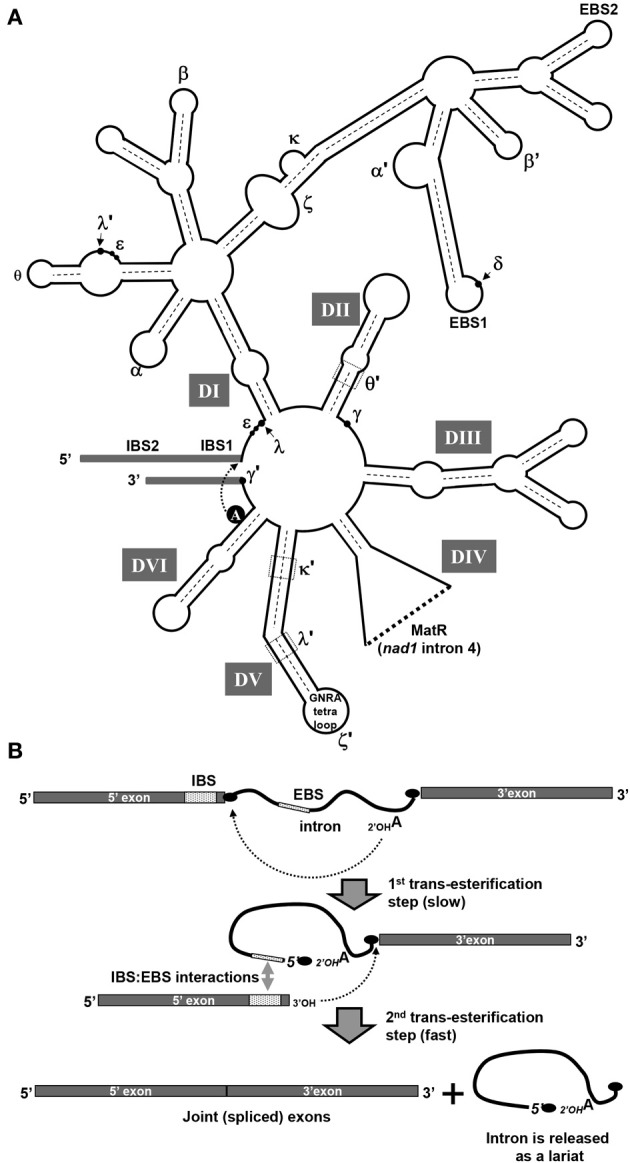
**Secondary structure model of plant mitochondrial group II introns. (A)** The secondary structure of group II introns is characterized by six double-helical domains (I-VI), arising from a central hub. Each subdomain of DI and DII, DIII, DIV, DV, and DVI are outlined within the structure. All plant mitochondrial intron structures in angiosperms are classified as standard group IIA RNAs (Bonen, 2008). The conserved bulged-A residue in DVI, the exon-intron binding sites (i.e., EBS1/IBS1 and EBS2/IBS2), and tertiary interactions between different intron regions (indicated by roman letters) are shown in the model structure. The ORF encoding the MatR protein in *nad1* intron 4 is encoded in intron domain IV. **(B)** Splicing pathway of the autocatalytic group II introns occurs by a two-step trans-esterification pathway. In the first step of the branching pathway, the 2'-OH group of the branch point adenosine nucleophilically attacks the phosphate at the 5'-splice site. The 5'-exon is released and the attacking adenosine adopts a 2',5'-branched structure that gives the intron a lariat form. Yet, in addition to this classical “branch-point” splicing reaction, some mitochondrial introns which lacks a DVI bulged A are excised as linear molecules which are generated by a “hydrolytic-pathway” of splicing (Li-Pook-Than and Bonen, 2006).

Introns within organellar genomes in plants belong to group I and group II (Michel et al., 1989; Bonen and Vogel, 2001; Zimmerly et al., 2001; Lambowitz and Zimmerly, 2004; Bonen, 2008). The vast majority of mitochondrial introns in angiosperms are classified as group II intron RNAs (Bonen, 2008). Introns in this class have been identified in prokaryotes, where they are relatively rare, and in the mitochondria of fungi, protists and a few primitive metazoans and in chloroplasts. Group II introns are particularly prevalent, however, in the mtDNAs in vascular plants (Malek and Knoop, 1998; Bonen and Vogel, 2001; Belfort et al., 2002; Bonen, 2008; Lambowitz and Zimmerly, 2011). These large introns are defined mainly by their capacity to fold into a conserved secondary structure of six domains (DI–DVI) extending from a central hub (Michel and Ferat, 1995; Qin and Pyle, 1998). Such intron structure models were later supported by a crystal structure of a self-spliced group II intron from *Oceanobacillus iheyensis*, further showing that multiple interactions between the different domains are indeed required to stabilize the tertiary structure of group II introns into their catalytically active forms (Toor et al., 2008). Figure 2A represents a secondary structure model of plant mitochondria group II intron RNAs.

### Splicing mechanism

The splicing of group II introns is mechanistically identical to that of the nuclear spliceosomal introns. Splicing is initiated through a trans-esterification involving a nucleophilic attack on the 5' splice site by the 2' hydroxyl group of an adenosine residue in domain VI (see Figure 2B). This results in the formation of a free 3' hydroxyl at the 5' splice site and a lariat shaped intermediate. In a second trans-esterification step, the flanking exons are joined when the free 3' hydroxyl at the 5' splice site forms a 5'-3' phosphodiester bond with the nucleotide at the 3' splice site; the intron is then finally released as a lariat. An alternative splicing reaction involves the “hydrolytic pathway,” where a water molecule acts as the nucleophile in the first step and cleaves the 5'-exon from the intron without forming a branched structure (Daniels et al., 1996). The second step of splicing is identical to the “branched-point” reaction; the 3'-terminal OH group of the 5'-exon attacks the phosphate at the 3'-splice site, thereby splicing the exons and releasing the intron as a linear form. The presence of both linear and circular forms of excised intron molecules in plant mitochondria points to multiple novel group II splicing mechanisms *in vivo* (Li-Pook-Than and Bonen, 2006).

Because some group II introns can splice auto-catalytically, their splicing, and by extension the splicing of nuclear spliceosomal introns, can be considered to be an example of RNA-mediated catalysis. The conditions required for *in vitro* splicing are generally non-physiological and most, if not all, group II introns require proteins for splicing *in vivo.*

Few organellar introns, and the majority of prokaryotic group II introns, can function as “homing” retroelements that insert themselves into related genomic sites (Cousineau et al., 1998; Lambowitz and Zimmerly, 2004). This process is mediated through the association of the intron with a specific reverse transcriptase (RT) encoded within the domain IV of the intron itself, termed as maturases (see below and Figure 2). In general, the interaction between the maturase and its cognate group II intron is critical for both splicing and mobility (Lambowitz and Zimmerly, 2004).

Interestingly, many plant organellar group II introns lack an ORF capable of specifying a maturase, or they encode a degenerate maturase protein which is probably unable to promote intron mobility (Michel et al., 1989; Bonen and Vogel, 2001; Zimmerly et al., 2001; Barkan, 2004; Lambowitz and Zimmerly, 2004; Bonen, 2008). These introns must therefore rely on other cofactors to facilitate their splicing *in vivo* (see below). Group II introns have not yet been identified in the nuclear genomes of eukaryotes, but their predicted descendants (i.e., the spliceosomal introns and retrotransposons), are highly abundant in eukaryotes, comprising together more than half of the genome in mammals.

### Cis- and trans-splicing

In prokaryotes, group II introns are often found near genes or after *rho* elements (Dai et al., 2003), and are thus expected to be expressed at low levels and to have only little effect on bacteria fitness. By contrast, the mitochondrial introns in plants are commonly (if not exclusively) found within the coding regions of various genes required for both genome expression and energy transduction (Bonen and Vogel, 2001). Structure-wise, some of the organellar introns in plants have diverged considerably from their bacterial ancestors and have lost elements considered to be essential for splicing (Bonen and Vogel, 2001; Bonen, 2008). Accordingly, none of the mitochondrial introns in angiosperms has been found to self-splice *in vitro*. Moreover, several of the mitochondrial introns in plants (i.e., *nad 1* introns 1 and 3, *nad2* intron 2, and *nad5* introns 3 and 4; Table 1) are fragmented in that they are transcribed as separate RNAs, which must assemble *in trans* through base-pairing interactions to form a splicing-competent structure (Chapdelaine and Bonen, 1991; Knoop et al., 1991; Pereira de Souza et al., 1991; Malek and Knoop, 1998; Bonen and Vogel, 2001; Bonen, 2008). In addition to *nad1, nad2*, and *nad5*, fragmentation of group II introns can be also observed in various other introns, as in the cases of the fourth intron in *nad1* in tobacco and rice and *cox2* intron in onion (Bonen, 2008; Kim and Yoon, 2010). The *trans*-spliced group II introns in plants are typically bipartite in structure, with their fragmentation sites occurring within the introns domain IV. Interestingly, this situation is reminiscent of the *trans-*interaction of snRNAs of the spliceosome with substrate pre-mRNAs, which replicates certain features of the group II introns (Sharp, 1985; Pyle and Lambowitz, 1999). This is discussed in greater detail below.

**Table 1:** **Mitochondrial group II introns and their identified splicing factors in higher plants**.

**Intron**	**Comments^a^**	**Splicing factor(s)**	**References**
(*nad1* intron 1)^b^	trans-spliced; lacks a bulged A residue in domain VI^c^	OPT43	Falcon de Longevialle et al., 2007
		nMAT1	Keren et al., 2012
*nad1* intron 2	degenerate; no clear bulged A residue in domain VI^c^	nMAT2	Keren et al., 2009
		mCSF1	Zmudjak et al., 2013
(*nad1* intron 3)	trans-spliced	mCSF1	Zmudjak et al., 2013
*nad1* intron 4			
*nad2* intron 1	lacks a bulged A residue in domain VI^c^	nMAT1	Keren et al., 2012
		mCSF1	Zmudjak et al., 2013
		mTSF1?^d^	Haïli et al., 2013
(*nad2* intron 2)	trans-spliced	RUG3	Kühn et al., 2011
		mCSF1	Zmudjak et al., 2013
		mTSF1?^d^	Haïli et al., 2013
*nad2* intron 3	degenerate intron	RUG3	Kühn et al., 2011
		ABO5	Liu et al., 2010
		mCSF1	Zmudjak et al., 2013
*nad2* intron 4		mCSF1	Zmudjak et al., 2013
*nad4* intron 1		NMS1^e^	Brangeon et al., 2000
*nad4* intron 2	degenerate intron; lacks a bulged A residue in domain VI^c^	nMAT1	Keren et al., 2012
*nad4* intron 3			
*nad5* intron 1		mCSF1	Zmudjak et al., 2013
(*nad5* intron 2)	trans-spliced; degenerate intron	mCSF1	Zmudjak et al., 2013
(*nad5* intron 3)	trans-spliced; degenerate intron	mCSF1	Zmudjak et al., 2013
*nad5* intron 4		mCSF1?^d^	Zmudjak et al., 2013
*nad7* intron 1		BIR6	Koprivova et al., 2010
*nad7* intron 2		nMAT2	Keren et al., 2009
		mCSF1	Zmudjak et al., 2013
*nad7* intron 3			
*nad7* intron 4			
*ccmFc*		WTF9	Colas des Francs-Small et al., 2012
*cox2*		nMAT2	Keren et al., 2009
		PMH2^f^	Köhler et al., 2010
		mCSF1	Zmudjak et al., 2013
*rpl2*	degenerate intron; no clear bulged A residue in domain VI^c^	WTF9	Colas des Francs-Small et al., 2012
*rps3*	degenerate intron; no clear bulged A residue in domain VI^c^	mCSF1	Zmudjak et al., 2013

a Data modified from Bonen (2008).

b Brackets indicate trans-spliced introns.

c Introns lacking a bulged adenosine (A) that acts as the nucleophile in the first trans-esterification step during splicing (see Bonen, 2008)

d Mutants are only partially affected in the splicing.

e A nuclear mutation in Nicotiana sylvestris; the identity of NMS1 is currently unknown.

f In addition to cox2, the efficiencies of many other splicing events and the steady-state levels of several mature mRNAs are reduced in the pmh2 mutants. Such non-specific RNA binding activity is concordant with RNA-chaperones, which facilitate transitions from non-functional to active conformations of structured RNAs (Köhler et al., 2010).

### Mis-splicing

**Figure 3:**
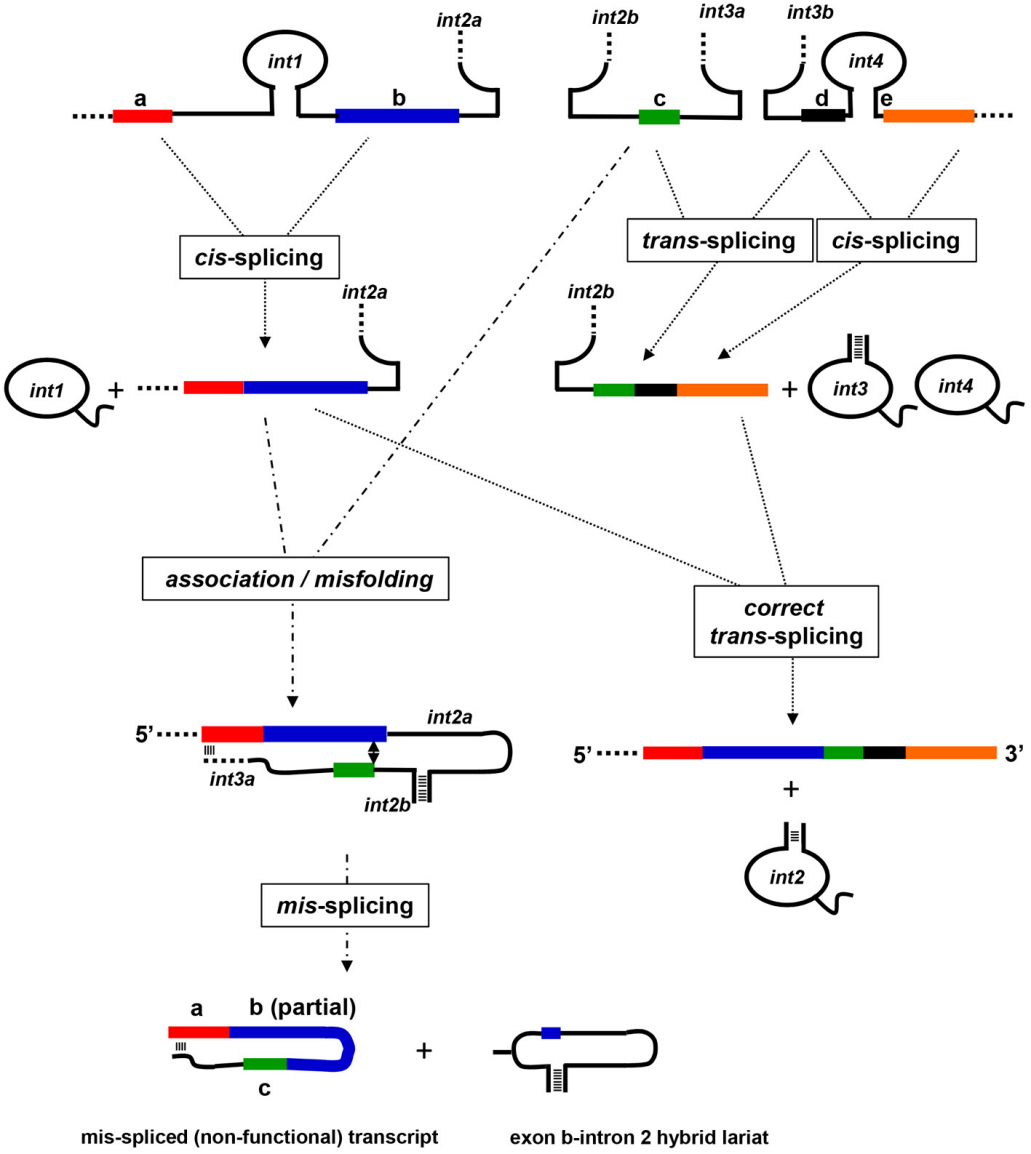
**Model for mis-splicing of mitochondrial group II introns**. A schematic illustration of a model for the splicing and mis-splicing of angiosperm *nad5* transcripts based on the model of Elina and Brown (2010). The *cis*-splicing of introns 1 and 4 proceeds normally. If the *trans*-splicing intron 3 assembles prior to the assembly of the *trans-*splcing intron 2, it splices correctly, and intron 2 can assemble and splice correctly to generate a properly spliced and functional mRNA. If intron 2 assembles first, however, a sequence at the 3' end of intron 3a (the portion of the intron co-transcribed with exon c) base pairs with a sequence within exon a, preventing correct positioning of the 5' splice site of intron 2. This results in the joining of exon c to sites within exon b to form mis-spliced, non-functional transcripts composed of exon a, a portion of exon b, exon c, and intron 3a; these transcripts do not engage in further splicing. A hybrid lariat mis-splicing product composed of intron 2 and a portion of exon b is also formed.

The *nad5* gene in most angiosperms contains four group II introns, of which the second and third flank a small, 22 bp exon and splice in “*trans*.” The formation of a mature *nad5* mRNA therefore involves the production of three independently transcribed transcripts and two distinct RNA-RNA associations through which the functional second and third introns assemble and splice. Analysis of partially spliced products generated when only one of the two trans-splicing events had occurred, revealed that these reactions must take place in a particular sequence (see Figure 3). When the splicing of the third intron precedes that of the second, a properly *trans*-spliced intermediate is formed that is competent to correctly engage in the other *trans*-splicing event. If the two portions of the second intron associate prior to the removal of the third intron, however, in a number of monocot and dicot plants, including plants of the *Arabidopsis, Brassica, Beta, Zea*, and *Triticum* genera, a variety of mis-spliced products are generated in which the correct 3' splice site is joined to any of a number of sites present in exon b (Elina and Brown, 2010). A model to explain these surprising results was formulated based on the observation that the 3' end of the portion of the third intron co-transcribed with exon c contained a sequence that was potentially capable of forming an extended duplex with the first exon. It was proposed that when the initial base-pairing interactions between the two portions of the second intron occurred, the sequences from the third intron and exon a annealed. This interaction sterically hindered the 3' splice site from assuming a position where it could join the correct 5' splice site; instead the 3' splice site joined to a variety of alternative sites within the upstream exon.

In plants of the genus *Oenothera*, the third *nad5* intron is further split, and requires the assembly of three separate transcripts to form a splicing-competent form (Knoop et al., 1997). In this case, the portion of the third intron predicted to base pair with the first exon “a” is not present on the same RNA as the 3' splice site of the second intron. Accordingly, mis-splicing of the second intron would be predicted to not take place, and this expectation was confirmed through the analysis of partially spliced products. Thus, the problem of intron 2 mis-splicing that was generated during the first evolutionary fragmentation of intron 3 was resolved through a second fragmentation. As discussed below, these findings may provide some insight into the evolutionary pressures that may have driven the evolution of the spliceosome from a group II intron in early evolving eukaryotes.

## Group II intron splicing factors

Proteins which facilitate the splicing of group II introns have probably originated in two ways (Lambowitz et al., 1999; Barkan, 2004; Fedorova et al., 2010): some are encoded within the introns themselves (Intron Encoded Proteins, or maturases) and have ancient relationships with their host introns, whereas others are derived “more recently” from the nuclear genome.

### Maturases

Maturases are characterized by three functional domains, which are required for both splicing and intron mobility activities: an N-terminal RT that is related to HIV-RTs, followed by an RNA-binding and splicing motif (domain X) and a site-specific endonuclease (D/En) motif at the C-terminus. Genetic and biochemical data show that the processing of group II introns in bacteria is facilitated by a single maturase-ORF encoded within DIV of the intron itself (see Figure 2A), although in some cases the maturase may also act on additional closely-related RNAs. The best characterized maturase to-date is LtrA protein, which binds with high affinity and specifically to its own intron, the self-splicing *Ll.LtrB* intron (Wank et al., 1999; Matsuura et al., 2001; Noah and Lambowitz, 2003). These assays indicated that the binding of LtrA facilitates *LtrB* splicing and folding under physiological conditions and is required for intron retrohoming.

#### MatR

**Figure 4:**
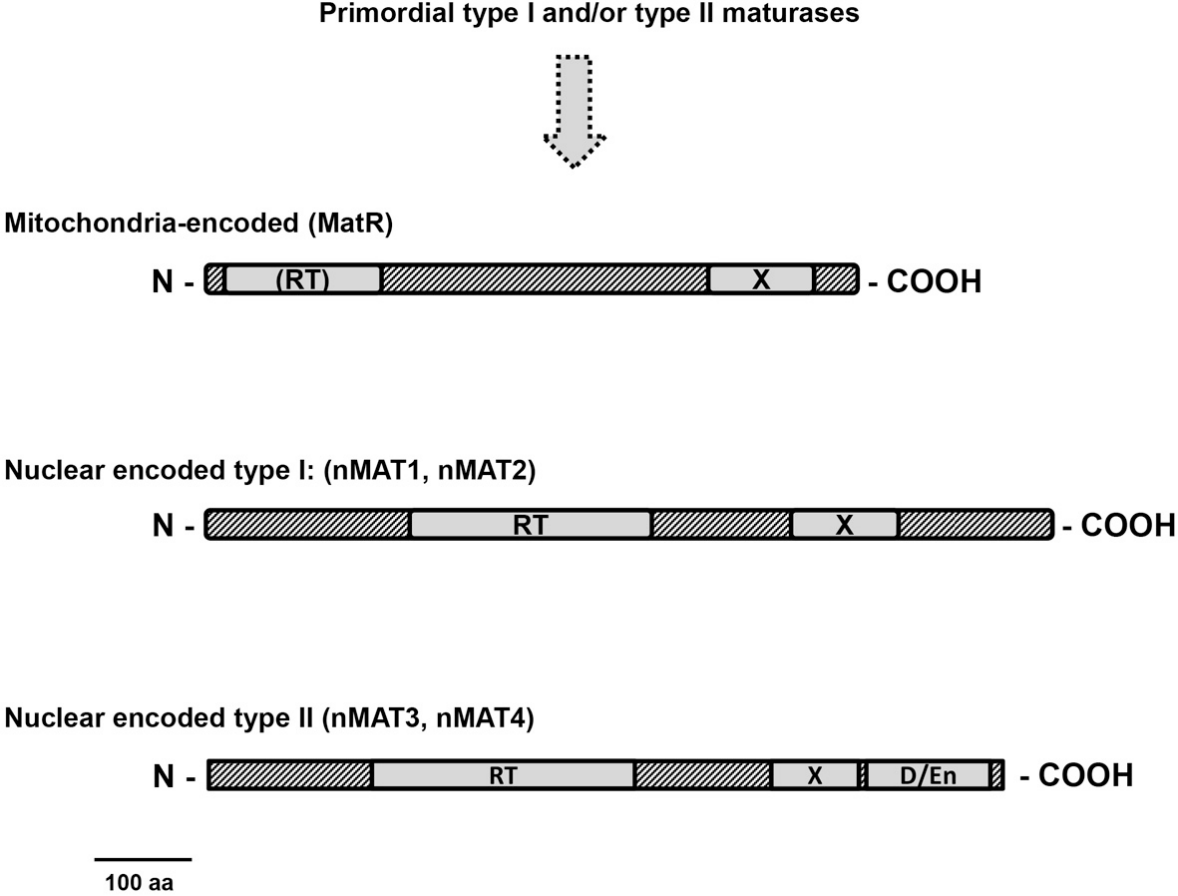
**Plant mitochondria maturases**. Plant maturase ORFs are shown as rectangles, with different shadings indicating conserved regions. The three domains typical to group II intron encoded maturases are outlined in the maturase ORFs: A reverse transcriptase (RT); domain X, associated with maturase activity; D, DNA-binding and endonuclease domain (D/En). The organelle-encoded MatR and MatK proteins contain a well conserved domain X, but have degenerate RT motifs and lack the En domain. In angiosperms, four other maturase-related proteins, denoted as nMAT 1–4, are encoded by nuclear genes, but have N-terminal mitochondrial targeting sequences (Keren et al., 2009). Similarly to MatR, the four nMATs have a conserved domain X, but show deviations in the RT domain and lack (type I) or have deviations in the D/En domain (type II), suggesting loss of mobility functions.

As outlined above, group II introns have diverged considerably from bacteria and plant organellar genomes. These introns in plants sometime lack regions which are considered to be essential for their splicing. Also, the number of maturases has been dramatically reduced during the evolution of organellar genomes in plants. The mitochondrial genomes of bryophytes, as *Marchantia polymorpha* and *Physcomitrella patens*, contain a few maturase ORFs, while only a single intron has retained its maturase gene in the mtDNAs in angiosperms: the *matR* gene encoded within *nad1* intron 4 (Wahleithner et al., 1990) (Figures 1,3). MatR proteins contain a well-conserved domain X, but have degenerate RT motifs and lack the En domain (Figure 4). The high conservation of MatR across the plant lineage (Adams et al., 2002) and RNA-editing events which restore conserved amino-acids (Thomson et al., 1994) suggest that *matR* encodes a functional protein. Preliminary data suggest that MatR binds to several group II introns *in vivo*, but its putative roles in splicing are yet to be determined. Analogously, MatK protein, encoded within *trnK* gene in chloroplasts, is associated with numerous group II introns *in vivo* (Zoschke et al., 2010). Besides MatR there are no splicing factor candidates among the ORFs of angiosperm mitochondrial genomes (although some lower plants may contain a few maturase ORFs in the mtDNAs). The splicing of mitochondrial introns in plants is therefore expected to be facilitated by nuclear-encoded proteins. These are translated by cytosolic ribosomes and subsequently imported into the organelle.

#### Nuclear-encoded maturases (nMATs)

In addition to *matR*, plants also contain several genes designated *nMat* 1 to 4, which are closely related to group II intron-encoded maturases and exist in the nucleus as self-standing ORFs, out of the context of their evolutionary related group II intron hosts (Mohr and Lambowitz, 2003; Keren et al., 2009). These are all predicted to reside within mitochondria and are thus expected to function in the splicing of organellar introns in plants. GFP-fusion analyses further established the mitochondrial localization of the four nuclear-encoded maturases *in vivo* in Arabidopsis, but also indicated a possible dual localization (in chloroplasts and mitochondria) for nMAT4 (Keren et al., 2009). Based on their topology and predicted evolutionary origins, the four nMATs are classified into two main groups (Figure 4): nMAT1 and nMAT2 are classified as type-I maturases, which lack the D/En motif, while nMAT3 and nMAT4 belong to type-II and contain all three domains (i.e., RT, X, and En/D) typical to “model” group II intron maturases (Mohr and Lambowitz, 2003). Yet, while the RT regions in nMAT1 and nMAT2 are degenerate, the D/En domains in nMAT3 and nMAT4 have mutations that are expected to inactivate the endonuclease activity (Mohr and Lambowitz, 2003). Thus, although expected to retain their splicing activities, the four nuclear-encoded maturases in angiosperms seem to have lost their mobility-associated functions.

Genetic studies indicate that nMAT proteins function in the splicing of mitochondrial introns in angiosperms (Nakagawa and Sakurai, 2006; Keren et al., 2009, 2012) and Table 1). In Arabidopsis, they seem particularly important for the maturation of primary *nad1* transcripts (see Table 1). Homozygote *nmat* mutants show altered growth and developmental phenotypes, modified respiration and altered stress responses, which are tightly correlated with mitochondrial complex I defects (Keren et al., 2009, 2012). While nMAT1 is required in *trans*-splicing of *nad1* intron 1, *nad2* intron 1, and *nad4* intron 2 (Keren et al., 2012), nMAT2 functions in the efficient splicing of *nad1* intron 2, *nad7* intron 1 and the single intron in the cytochrome oxidase subunit 2 gene (*cox2* intron 1) (Keren et al., 2009). Interestingly, the three intron targets of nMAT1 are all lacking the canonical bulged A residue, which is required for the first trans-esterification step and the release of the 5'-exon (see above and Figure 2). The precise biochemical functions of nuclear-encoded maturases in the splicing process have not yet been established, but an intriguing possibility is that nMAT1 may function in the hydrolysis of the phosphodiester bond at the 5' splice site, or recruit specific RNA nucleases required for the release of the 5'-exon. Similar to nMAT1 and nMAT2, studies in progress suggest that MatR nMAT3 and nMAT4 also play a role in the splicing of mitochondrial introns.

#### Maturase phylogeny

**Figure 5:**
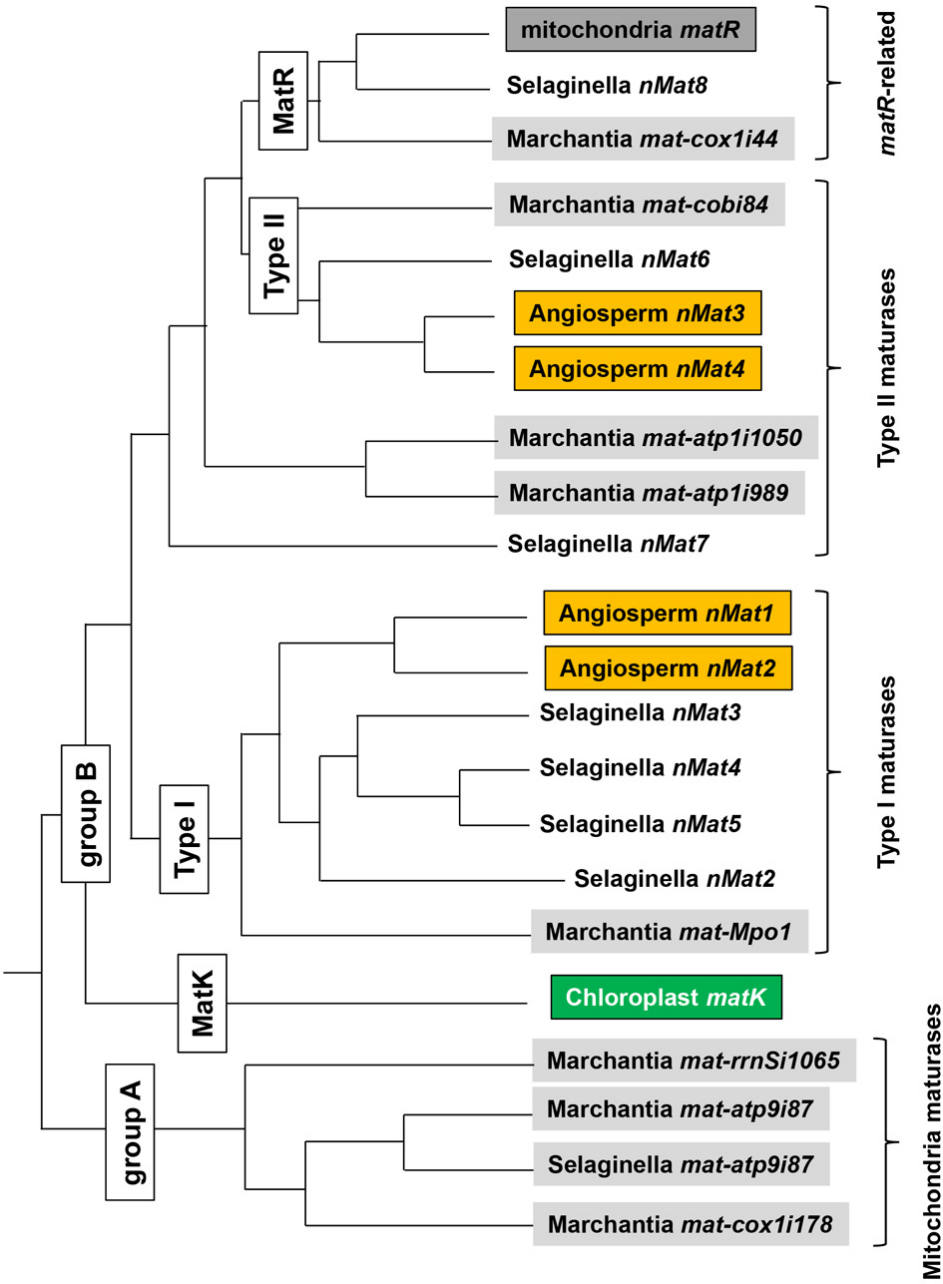
**A comparative sequence analysis of plant maturases**. Phylogentic relationships among seed plant, *Selaginella* and *Marchantia* mitochondrial, chloroplast and identified nuclear maturases, based on Guo and Mower (2013). Nomenclature of Guo and Mower (2013) is used for mitochondrial maturases other than *matR* (designated by the authors as *mat*-*nad1i728*). Nomenclature for nuclear maturases (*nMats*) is identical with that employed in this review and previous analyses (Mohr and Lambowitz, 2003; Keren et al., 2009). Branch lengths, as illustrated, do not represent phylogenetic distances and are intended only to illustrate relationships among orthologous *mat* genes in plants. Mitochondrially encoded genes are shown as gray shaded boxes and chloroplast *matK* is in green, while orange shaded boxes represent plant maturase homologs.

A recent study by Guo and Mower (2013) has provided new insights into the distribution and the evolution of land-plant maturases. The authors used known plant maturase sequences to conduct an extensive search of recently sequenced green algal and land-plant mitochondrial genomes, including those of the sequenced nuclear genomes of the lycophyte *Selaginella* and the moss *Physcomitrella*. The authors detected multiple, previously unrecognized plant sequences potentially cable of specifying a protein with maturase function. These included seven new nuclear maturase loci in *Selaginella* (bringing the total number of maturases for this species to eight), and five new nuclear maturase loci in *Physcomitrella* (bringing its total to 12). Building upon this new information a comprehensive phylogeny was constructed to determine the evolutionary relationships among the different maturase sequences; a simplified version of this phylogeny, displaying only the relationships among the seed plant (six maturase genes), *Marchantia* (eight related genes) and *Selaginella* maturases, is shown in Figure 5. Most, but not all of the new nMATs are predicted to be mitochondrial proteins.

Several interesting features of plant maturase evolution are revealed by this phylogeny. In general, plant *mat* genes fall into two main categories (Figure 5): mitochondria-encoded maturases which have no homologs in other plants (group A) and orthologous maturase-genes, found in the mitochondrial and/or nuclear genomes of different plants (group B). These can be further divided into four groups (i.e., *matR* sequences in land plants, type I and type II maturases and plastidial *matK* ORF in *trnK* intron). It is likely, therefore, that the angiosperm type I nMat1 and nMat2 genes resulted from a nuclear gene duplication, following the translocation to the nucleus of a single mitochondrial maturase related to Marchantia maturase mat-Mpo1 (formerly designated rtl, for RT-like) (Figure 5). It is further likely that three gene duplication events in the Selaginella lineage following the same translocation event led to four of the Selaginella nMats. As many mitochondrial genes, which were copied to a nuclear genome, the “original” mitochondrion-encoded Mpo1 ORF probably degenerated rapidly, resulting in the gene persisting only in the nucleus. Similarly, the type II nMat3 and nMat4 genes in angiosperms likely arose from a gene duplication event following the transfer to the nucleus of a maturase resembling modern Marchantia mat-cobi84 (see Figure 5); this event also resulted in the single Selaginella nuclear maturase nMat6. Interestingly, the matR maturases appear most similar to the Marchantia mat-cox1i44, suggesting common matures ancestor for these two maturases. Of particular interest is the observation that matK, the single maturase encoded in the chloroplast genome, clusters with mitochondrial maturases from group B, suggesting the possible transfer of a mitochondrial intron to the chloroplast in an ancestor of modern land plants and charophyte algae. Although branch support for the position of the matK clade was considered weak (Guo and Mower, 2013), this possibility was also suggested in an earlier analysis of matK phylogeny (Hausner et al., 2006).

### Host-encoded factors in plant mitochondria

Different RNA-binding proteins were shown to function in the splicing and processing of introns in plant organellar genomes (see below). Some, such as a DEAD RNA-helicase (PMH2) and a CRM-domain protein (mCSF1) are required for optimal splicing of a large set of introns, whereas others (e.g., PPR, PORR, and RCC proteins) rather appear to be specific for a single, or few, individual introns. Although this review focuses mainly on maturases, this section summarizes the information available about other types of mitochondrial group II intron splicing factors.

#### CRM

CRM (Chloroplast RNA splicing and ribosome Maturation) proteins are characterized by a conserved RNA-binding domain of ~10 kDa (Pfam-PF01985), which is related to a conserved bacterial ORF (YhbY) (Barkan et al., 2007). In *E.coli*, YhbY is mainly associated with the maturation of the pre-50S ribosomal subunit (Barkan et al., 2007). In eukaryotes, CRMs are restricted to the plant lineage, where they are found in a small family of proteins containing between 1 and 4 repeats of the conserved CRM domain (Barkan et al., 2007). Biochemical analyses indicated that CRM domains share structural similarities and RNA-binding characteristics with the RNA recognition motif (RRM) (Keren et al., 2008). The nuclear genomes in angiosperms contain ~15 CRM proteins, of which the majority are predicted to be plastidial by different targeting prediction programs, although few may exist within the mitochondria and nucleus as well (Barkan et al., 2007). Two of these paralogs in Arabidopsis, mCSF1 and mCSF2, are targeted to mitochondria *in vivo* (Zmudjak et al., 2013). Genetic and biochemical data indicate that mCSF1 encodes an essential polypeptide which is required for the processing of many of the mitochondrial introns in Arabidopsis (Zmudjak et al., 2013). Accordingly, *RNAi*-knockdown *mcsf1* lines show strong defects in the assembly of both complex I and complex IV (Zmudjak et al., 2013). No other mitochondrial CRM domain protein has been described so far, but other mitochondrial CRM members are expected to have important roles in RNA metabolism in higher plant mitochondria.

#### RNA DEAD-box helicases

RNA helicases from the DEAD-box family are widely distributed in both prokaryotes and eukaryotes and have essential roles in RNA processing. These include ATP-dependent RNA duplex unwinding and/or formation, displacement of proteins from RNA transcripts, formation of assembly platforms for larger ribonucleoprotein complexes, and also the sensing of bacterial metabolites (Putnam and Jankowsky, 2013). These processes are fundamental steps in RNA metabolism in all organisms. About 60 such DEAD-box encoding genes have been identified in plants, but most have not yet been characterized. One of these members in Arabidopsis, PMH2 (Putative Mitochondrial Helicase 2), is found in a large ribonucleoprotein complex *in vivo*, and is thought to be a hetero-multimeric splicing unit (Matthes et al., 2007; Köhler et al., 2010). Although the activities of PMH2 are not essential for the maturation of the mitochondrial pre-mRNAs, homozygous *pmh2* mutants in Arabidopsis are affected in the splicing efficiency of many organelle introns, as evident by increased levels of many pre-mRNAs in *pmh2* mutant mitochondria.

#### PPR proteins

PPR proteins (Small and Peeters, 2000) are found in most eukaryotes, but are in particularly abundant in plants, with nearly 500 different members the nuclear genomes of both monocot and dicot species (Lurin et al., 2004). Many, if not all, of the characterized PPR proteins in plants were shown to be involved in post-transcriptional RNA processing events in mitochondria and chloroplasts, including RNA editing, trimming, RNA stability, and more recently also in the removal of introns (Schmitz-Linneweber and Small, 2008). PPR proteins which function in the splicing of mitochondrial pre-mRNAs include OTP43 (Falcon de Longevialle et al., 2007), BIR6 (Koprivova et al., 2010), and ABO5 (Liu et al., 2010), all dealing with transcripts encoding subunits of the respiratory complex I (see Table 1).

Based on their topology and number of repeats, the PPR family is divided into two major classes, P and PLS (Lurin et al., 2004). In angiosperms, the splicing factors, together with several other PPR proteins which function in RNA stability and protection, belong to the P subclass and identified as “pure” PPR proteins, while editing factors are generally PLS domain proteins containing additional C-terminal domains (Schmitz-Linneweber and Small, 2008). Other P-class factors, which are predicted to reside within mitochondria, are therefore anticipated to function in the splicing of mitochondrial pre-mRNAs. However, PPR proteins belonging to the PLS class may also be involved in group II intron splicing in angiosperm mitochondria, as shown in Arabidopsis chloroplasts (OTP70, E subfamily) (Chateigner-Boutin et al., 2011) and the splicing of the mitochondrial *cox1* transcript in *Physcomitrella patens* by PpPPR_43 (a DYW subclass PPR protein) (Ichinose et al., 2012). Recently, the code of RNA recognition by PPR proteins was deciphered (Barkan et al., 2012; Yagi et al., 2013), and was further established by structural data (Yin et al., 2013). Accordingly, systematic bioinformatics studies provide a powerful tool for molecular characterization of the roles additional PPRs in mitochondria RNA metabolism in plants.

#### PORR domain family

In addition to known RNA-binding proteins, genetic studies also led to the identification of novel factors which function in the processing of group II introns in plant organelles. Among these is the PORR (Plant Organellar RNA Recognition) domain which is represented in a small family of proteins in angiosperms (15 in Arabidopsis and 17 in rice) (Kroeger et al., 2009). One of these members in maize is associated with different group II introns *in vivo* and was shown to promote the splicing of about half of the plastid introns (Kroeger et al., 2009). In contrast, a mitochondrial member of the family, WTF9, is more specific and was shown to act on two group II introns, namely the essential ribosomal *rpl2* subunit and *ccmF*_*C*_ (encoding a subunit of the *c-*type cytochrome maturation system) (Colas des Francs-Small et al., 2012). Interestingly, homozygous Arabidopsis *wtf9* lines are viable, possibly because the *rpl2* transcript has been “split” during its evolution and is encoded in two parts, one of which is nuclear- encoded in Arabidopsis (Colas des Francs-Small et al., 2012). Thus, the lack of splicing of the mitochondrial *rpl2* transcript leads to a partially functional Rpl2 protein. The strong phenotypes of the *wtf9* mutants were therefore mainly attributed to the depletion of cyt*c* and cyt*c*_1_, and subsequently to complex III and complex IV defects (Colas des Francs-Small et al., 2012).

#### RCC proteins

Another factor involves a eukaryotic protein (RCC1, Regulator of Chromosome Condensation) which binds to chromatin and interacts with the nuclear Ran GTPase (Dasso, 1993). Similarly to PPRs, RCC proteins also contain tandem repeats of a conserved domain of about 50 amino acids. The association of RCC1 with Ran is postulated to be important in the regulation of nuclear gene expression. Only one member of the RCC1 domain family, RUG3, has been identified so far as a splicing factor. RUG3 is closely related to RCC1 and UV-B RESISTANCE 8, which were shown to function in chromatin modification. The RUG3 protein is involved in the splicing of *nad2* in Arabidopsis (Kühn et al., 2011). Similar to other complex I mutants, *rug3* knockout lines show slow growth and reduced size. As no RNA-binding was demonstrated for RCC1-like proteins, it has been proposed that these factors may recruit RNA-binding cofactors, such as the ABO5 protein (Liu et al., 2010; Kühn et al., 2011), to the splicing complex.

**Figure 6:**
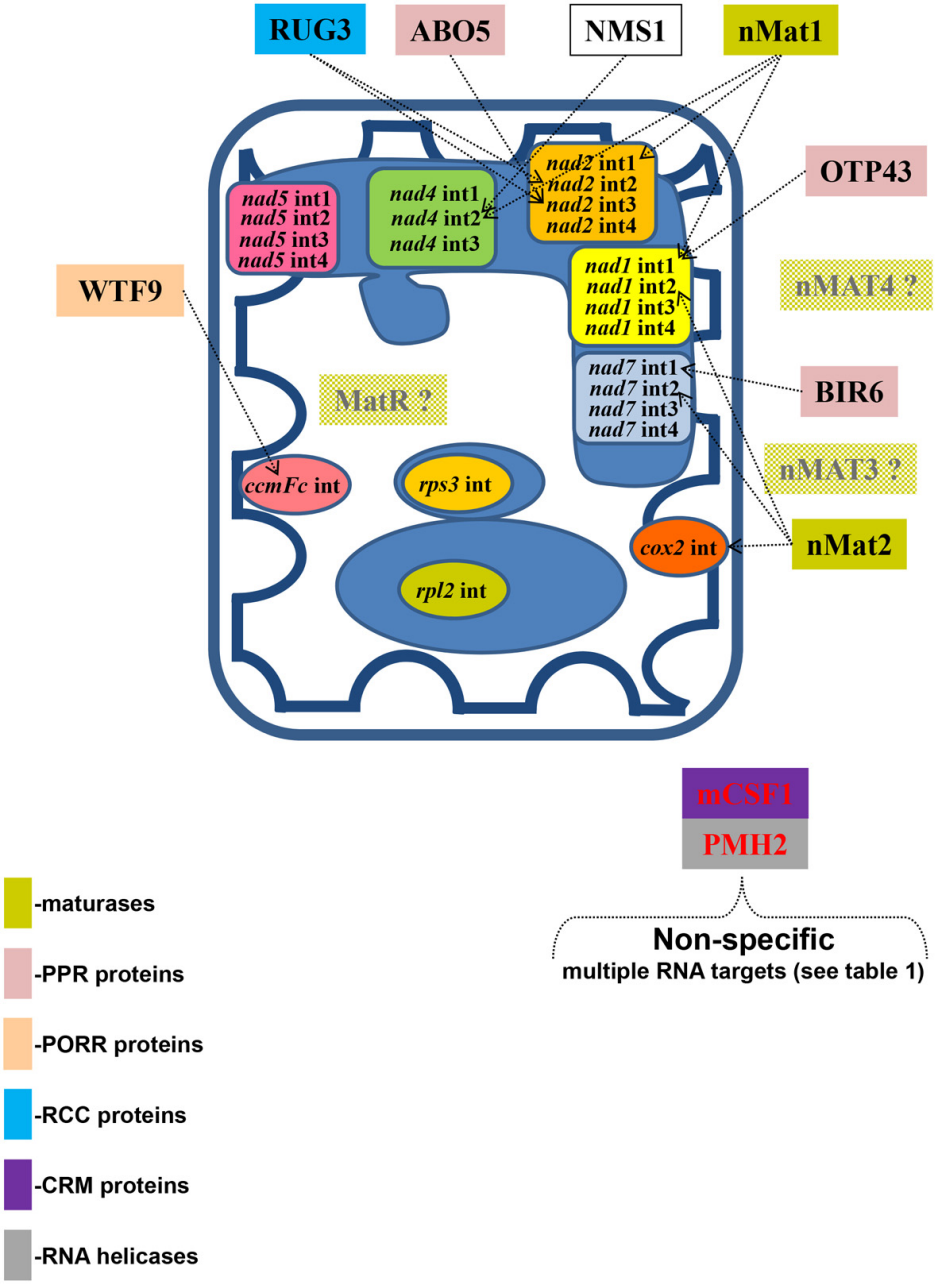
**The splicing of individual group II introns in Arabidopsis mitochondria is facilitated by multiple protein cofactors**. Genetic and biochemical data indicate that mitochondrial splicing factors are essential for organellar functions and thus for plant survival. These analyses, involving selected mutants in various nuclear-encoded mitochondrial splicing factors, also indicate that the processing of individual mitochondrial introns in plant is facilitated by multiple protein cofactors. The different mitochondrial introns in angiosperms and their specific splicing factors are indicated in the figure. Colors refer to different protein families, while the shaded boxes indicate preliminary biochemical and genetic data in Arabidopsis.

In addition to the identified factors, a nuclear mutation in *Nicotiana sylvestris* (*nms1*) was shown to disrupt the splicing of the first intron in the *nad4* transcript (Brangeon et al., 2000), however the identity of this gene remains unknown. The specific roles of these factors in the splicing and processing of mitochondrial group II introns are illustrated in Figure 6.

## Physiological consequences of mitochondrial group II intron splicing defects

The challenges of maintaining prokaryotic-type structures and functions in the cell are common to all eukaryotes. The respiratory chain is composed of four major complexes, complexes I to IV, which are localized within the mitochondrial inner-membrane. These complexes contain subunits encoded by both nuclear and mtDNAs. A miscommunication between the organelles and their host cell (i.e., a breakdown in the nuclear-organellar “cross-talk”) often results in severe developmental defects.

Plants possess some of the most complex organelle compositions of all known eukaryotic cells. As sessile organisms, land-plants must cope with multiple environmental stresses. During their evolution, plants have acquired complex regulatory mechanisms to coordinate cellular functions with environmental stresses. The participation of nuclear-encoded factors in organelle RNA metabolism may therefore provide means to control the biogenesis of the respiratory (and photosynthetic) machineries, and thus to link cellular metabolism and environmental and developmental signals. Following this idea, the splicing of both plastidial (Barkan, 1989; Kahlau and Bock, 2008) and mitochondrial introns (Li-Pook-Than et al., 2004; Dalby and Bonen, 2013) is developmentally and environmentally responsive. Likewise, the expression of group II intron splicing factors and other genes implicated in mitochondrial genome expression and RNA metabolism in plants is tightly regulated during the early stages of germination (Narsai et al., 2011).

The importance of these stages is further reflected in plant mutants affected in genes required for the splicing of mitochondrial introns. Such mutants exhibit reduced germination and seedling establishment, and are often affected in growth and development (see Keren et al., 2012). As many of the introns in the mtDNAs in angiosperms are inserted in complex I subunit genes, but some also reside in *ccmFc, cox2* and the ribosomal *rpl2* and *rps3* genes, the splicing mutants are strongly affected in respiratory associated functions and cellular metabolism (Keren et al., 2012). These mutants are viable in plants, plausibly due to the presence of non-energy conserving respiratory pathways which allow electron transport from NAD(P)H to oxygen (O_2_) (reviewed in Millar et al., 2011). These pathways also allow plant mitochondria to respire in the presence of rotenone and cyanide, inhibitors of complex I and complex IV, respectively. Thus, the analysis of nuclear-encoded factors required in pre-mRNA processing is expected to give new insights into these processes in plant organellar biology and to contribute to our overall understanding of complex regulatory pathways controlling the biogenesis of respiratory apparatus in plants and other eukaryotic systems (Meyer et al., 2011; Colas des Francs-Small and Small, 2013).

## Perspectives

### Mitochondrial group II introns—progenitors of the nuclear spliceosome machinery?

Based on the structural features, the similarity of exon-intron boundaries and a common splicing-mechanism, group II introns are proposed to be ancestors of the eukaryotic spliceosomal introns (Sharp, 1985; Cech, 1986; Lynch and Kewalramani, 2003; Martin and Koonin, 2006). While bacterial group II introns are generally thought to be self-sufficient with respect to splicing, numerous different nuclear-encoded factors are required to support the splicing of organellar group II introns in angiosperms (de Longevialle et al., 2010; Barkan, 2011). While only a single intron maturase has been retained in the two organelle genomes of angiosperms, several other maturase genes have been transferred into the nucleus during the evolution of plants. These are all postulated to function in the splicing of organellar introns. Similarly, genetic screens in *Chlamydomonas reinhardtii* demonstrated that the *trans*-splicing of the two group II introns in its chloroplast genome, is also facilitated by a large set of nuclear-encoded RNA binding cofactors (Goldschmidt-Clermont et al., 1990; Perron et al., 2004). Recently, a group II intron in *Clostridium tetani*, was shown to undergo alternative splicing reactions resulting with five alternative mRNAs products, each encoding a distinct protein isoform (McNeil et al., in press). Together, these observations further suggested that a group II intron invader of the eukaryotic cell nucleus, possibly derived from the mitochondrial symbiont, served as the evolutionary precursor to the nuclear splicesomal introns. According to this view, the RNA structures required for group II catalysis began to function in “*trans*” in a primitive spliceosome, and the group II structural features of the target primordial introns degenerated. Interestingly, features of some plant mitochondrial introns reflect possible stages that may have occurred in such a process. Several undergo *trans*-splicing: different portions of the intron are assembled from two and in one case, three different transcripts. At least one *trans*-splicing intron undergoes a significant degree of mis-splicing, and this is lost in plants in which the intron is further fragmented, a process that may have played a role in the formation of the primordial spliceosome. Also, several introns lack key structural features, such as the bulged adenosine in the DVI stem, which serves as the nucleophile in the first splicing step (Figure 2) and may be reflective of an early stage in the structural degeneracy that took place in target introns of the evolving spliceosome.

Maturases and their accessory splicing factors in plant mitochondria, thus represent a highly versatile set of proteins which differ from their presumptive monospecific ancestors. These features may correspond to a further evolutionary link between mitochondrial group II introns and the spliceosomal machinery in nuclear genomes of most eukaryotic cells. Likewise, the recruitment of different protein cofactors to assist with the splicing of mitochondrial introns in plants (see Figure 6) may reflect processes analogous to those that took place during the evolution of the nuclear spliceosome. In particular, the transition of maturases from specific to versatile splicing factors, may have allowed them to serve as principal component in the evolution of the nuclear spliceosome with its ability to act on a distinct subset of introns in “*trans*.” The homology of group II maturases to the core spliceosomal splicing factor (Dlakic and Mushegian, 2011) further supports this view. Future research will be aimed at determining whether plant maturases have gained the ability to act on multiple intron targets by acquiring versatility in intron recognition and intron structure modification.

### Conflict of interest statement

The authors declare that the research was conducted in the absence of any commercial or financial relationships that could be construed as a potential conflict of interest.
